# Regulation of the Key Epithelial Cancer Suppressor miR-124 Function by Competing Endogenous RNAs

**DOI:** 10.3390/ijms232113620

**Published:** 2022-11-07

**Authors:** Eleonora A. Braga, Marina V. Fridman, Alexey M. Burdennyy, Elena A. Filippova, Vitaly I. Loginov, Irina V. Pronina, Alexey A. Dmitriev, Nikolay E. Kushlinskii

**Affiliations:** 1 Institute of General Pathology and Pathophysiology, 125315 Moscow, Russia; 2 Research Centre for Medical Genetics, 115522 Moscow, Russia; 3 Vavilov Institute of General Genetics, Russian Academy of Sciences, 119991 Moscow, Russia; 4 Engelhardt Institute of Molecular Biology, Russian Academy of Sciences, 119991 Moscow, Russia; 5 N.N. Blokhin National Medical Research Center of Oncology, 115478 Moscow, Russia

**Keywords:** epithelial cancers, epigenetic mechanisms, miR-124, lncRNA, circRNA, EMT, metastasis, signaling pathways, chemoresistance

## Abstract

A decrease in the miR-124 expression was observed in various epithelial cancers. Like a classical suppressor, miR-124 can inhibit the translation of multiple oncogenic proteins. Epigenetic mechanisms play a significant role in the regulation of miR-124 expression and involve hypermethylation of the *MIR-124-1/-2/-3* genes and the effects of long non-coding RNAs (lncRNAs) and circular RNAs (circRNAs) according to the model of competing endogenous RNAs (ceRNAs). More than 40 interactomes (lncRNA/miR-124/mRNA) based on competition between lncRNAs and mRNAs for miR-124 binding have been identified in various epithelial cancers. LncRNAs MALAT1, NEAT1, HOXA11-AS, and XIST are the most represented in these axes. Fourteen axes (e.g., SND1-IT1/miR-124/COL4A1) are involved in EMT and/or metastasis. Moreover, eight axes (e.g., OIP5-AS1/miR-124-5p/IDH2) are involved in key pathways, such as Wnt/b-catenin, E2F1, TGF-β, SMAD, ERK/MAPK, HIF-1α, Notch, PI3K/Akt signaling, and cancer cell stemness. Additionally, 15 axes impaired patient survival and three axes reduced chemo- or radiosensitivity. To date, 14 cases of miR-124 regulation by circRNAs have been identified. Half of them involve circHIPK3, which belongs to the exonic ecircRNAs and stimulates cell proliferation, EMT, autophagy, angiogenesis, and multidrug resistance. Thus, miR-124 and its interacting partners may be considered promising targets for cancer therapy.

## 1. Introduction

Recent studies have increasingly shown that less than 1.5% of the human genome genes encode proteins. Non-coding RNAs (ncRNAs) transcribed from non-protein-coding genes are involved in the regulation of all biological processes. Among them, microRNAs (miRNAs) and long non-coding RNAs (lncRNAs) are of the greatest importance. These two large classes differ from each other in length [1]. miRNAs, composed of up to 24 nucleotides, suppress the translation of protein-coding genes of wide functionality. It has been shown that miRNAs are involved in many cellular processes as regulators of homeostasis and their expression is highly conserved in various cells. The key role of miRNAs in carcinogenesis has also been revealed. miRNAs may be oncogenic, suppressor, or may have either function, depending on cancer localization [2,3].

miR-124, first discovered in mice, is a typical suppressor miRNA. This miRNA, found not only in mammals but also in worms, is notable not only for its conservation but also for the unusual decrease in expression associated with the development of the malignant transformation of various etiologies [3,4]. Three genes encode for this miRNA: *MIR-124-1* (8p23.1), *MIR-124-2* (8q12.3), and *MIR-124-3* (20q13.33). All of them harbor CpG islands. The genes encoding miR-124 in various cancer types undergo methylation, suppressing their expression [5,6,7].

The interaction between miRNA and mRNA of the target protein-coding genes involves the formation of the RNA-induced silencing complex (RISC) [8,9]. The 3′-UTR (3′-untranslated region) of mRNA sequences may contain sites for complementary binding of different miRNAs. RISC, together with a member of the Argonaute (AGO) protein family, provides complementary binding of miRNA to mRNA, which reduces protein expression through mRNA cleavage, degradation, and/or translational repression [8,9].

The lncRNA class of ncRNAs with a length of more than 200 nucleotides has multiple functionalities [10] and participates in the regulation of all cellular processes, including the expression of miRNAs themselves. Similar to miRNAs, the role of lncRNAs in cancer may be oncogenic, suppressor, or dual [11]. Noteworthy, the two ncRNA classes—miRNAs and lncRNAs—are in a complex interaction with each other. This interaction involves messenger RNAs and ncRNAs, such as lncRNA, pseudogenes, circular long ncRNAs (circRNAs), and miRNAs. mRNAs and ncRNAs interact with each other and co-regulate each other by competing for binding to shared miRNAs [12]. Hereby, ncRNAs act as competing endogenous RNAs (ceRNAs) and form interactomes in an ncRNA/miRNA/mRNA pattern. Such interactomes are involved in the carcinogenesis of multiple tumors. For example, a large study showed that the axis UCA1/miR-124/JAG1 is responsible for developing tongue cancer [13].

The search for new markers for diagnosing and predicting epithelial tumors remains the most important task of current molecular oncology. In this regard, miRNAs and lncRNAs involved in the regulation of cellular processes and detected in various cancer types are very promising candidates [14,15]. The same refers to circRNAs, which are a useful tool for diagnosis and the assessment of the quality of treatment. CircRNAs were first discovered in viroids and are annular single-stranded molecules without 5′ caps or 3′ poly(A) tails. CircRNAs demonstrate high conservation, stability, and expression level in cells. Recently, the association of these ncRNAs with the development of various cancer types, as well as their interaction with the other classes of ncRNAs has been discovered, necessitating a further study of circRNAs [15].

In this review, various aspects of the regulation of miR-124, the suppressor miRNA that interacts with both ncRNAs and its targets in various types of epithelial tumors, will be considered.

## 2. Protein-Coding Target Genes of miR-124 and Their Role in the Biological Processes Involved in the Carcinogenesis of Epithelial Tumors

miR-124 is a subject of interest for the study for several reasons, including its high conservativeness in various tissues of many organisms, as well as the fact that its abnormal expression is associated with cancer of various etiologies. miR-124 has been shown to be involved in the regulation of cell growth, differentiation, and development, while the disruption of this miRNA can lead to the development of malignant cell transformation, in particular, to cell cycle arrest, epithelial-mesenchymal transition (EMT), metastasis and resistance to chemotherapy. Therefore, this miRNA is considered a potential biomarker for developing new therapeutic strategies for treating tumors [16].

Several studies have shown the suppressive nature of miR-124 in cancers of various etiologies. Also, for several cancers, the ability to suppress the proliferation, invasion, and migration of tumor cells has been shown. It is noted that, depending on the process and ectopic location, miR-124 uses different targets to suppress a particular type of cancer, although in tumor cells, regardless of etiology, this miRNA is suppressed in various ways, including hypermethylation of genes encoding it. The *CBL* (Casitas B-lineage lymphoma; encodes ubiquitin ligase E3), *PDCD6* (programmed cell death 6), *ROCK1* (Rho-associated protein kinase 1), *SNAI1/2* (Snail family transcription repressors 1 and 2), *TWIST1/2* (Twist family BHLH transcription factors 1 and 2), *ASPP* (apoptosis-stimulating protein p53), *iASPP* (inhibitor of apoptosis-stimulating protein p53), *SPHK1* (sphingosine kinase 1), *NRP1* (neuropilin 1) genes are the previously discovered target genes of this miRNA in different types of cancer. All these genes show oncogenic properties affecting the development and progression of epithelial tumors [3,4].

Note that not only proteins encoding by miR-124 target genes are important in the development of cancer. To assess the picture of pathological transformation, one should consider the cellular signal pathways that trigger when various system processes are disrupted. In particular, the involvement of miR-124 in the process of suppressing tumor activity as part of a cascade of interactions within the Wnt/β-catenin pathway deserves attention. Thus, it was revealed that miR-124 with miR-340 cooperation participates in the specific suppression of the *SRGAP1* gene (SLIT-ROBO Rho GTPase Activating Protein 1). As a result, this leads to the suppression of the growth of the gastric cancer cell line. The *SRGAP1* gene is an oncogene that, according to several studies, is not only triggered in cancer cells of various etiologies but is also part of the Wnt/β-catenin pathway as the main regulator of the activity of this pathway. Alternatively, miR-124 suppresses the expression of this gene, resulting in the entire cascade “fade” [3,4].

Recent studies provide an opportunity for a more complete assessment of the role of miRNAs in the development of cancers of various localizations. A study by a group of Chinese scientists on the role of miR-124 in the development of malignant transformation of liver cells has revealed a new target. The study consisted of several stages. At the stage conducted on hepatocellular cancer stem cell lines, it was shown that miR-124, whose expression level was high, suppressed the activity of the *CAV1* (Caveolin-1) gene found using bioinformatic screening. A mutual regulation within this pair was also noted. Moreover, the results showed tumor progression and revealed an unfavorable prognosis for patients with such gene interaction. One of the possible reasons for this outcome was called resistance to the standard therapeutic agent in this type of cancer when suppressing miR-124 in tumor cells. The authors consider a decrease in resistance to the therapeutic agent and oncogene expression with an artificial increase in miR-124 expression to be an optimistic result. Similar results were obtained for colon cancer [17,18].

Noteworthy, several other target genes have been identified for liver cancer: *C/EBPa* (CCAAT enhancer binding protein α), which encodes an important transcription factor and is also regulated by miR-124 [19], *CRKL* (V-crk sarcoma virus CT10 oncogene homolog (avian)-like), which is involved in various cellular processes [20], *BIRC3* (Baculoviral IAP Repeat Containing 3) and some others, the suppression of whose expression by miR-124 reduced the proliferative activity of tumor cells, invasion, and migration [21,22,23].

According to some studies, one of the important characteristics of malignant neoplasms is uncontrolled cell growth. The results of a study of a colon cancer-specific marker of the *IQGAP1* gene (IQ motif containing GTPase activating protein) have shown that there is a mechanism for suppressing this process. Scientists have found that direct regulation through miR-124 significantly reduces the level of oncogene expression by stopping the growth of tumor cells. It is supposed to be interesting the role of this pair in the launch of the mentioned Wnt/β-catenin pathway [24].

Another important characteristic of cancer is progression. Identifying a marker that allows you to assess the metastatic potential of a tumor is extremely important for understanding the anatomy of any type of cancer. Thus, in assessing the metastatic potential of miR-124 in breast cancer, the *ABCC4* (ATP-binding cassette subfamily C member 4) target gene was identified. The miR-124/ABCC4 pair was associated not only with tumor progression but also with drug resistance [25].

When studying the metastatic potential of miR-124, our group obtained some convincing results indicating a high level of metastasis formation in the resection material in ovarian and breast cancers. In a study of 20 miRNA genes in ovarian carcinoma, we showed an association of aberrant methylation of several miRNA genes, including miR-124, with the subsequent development of metastases [26]. It has been shown that because of aberrant methylation, the entire regulatory cascade is disrupted, which leads to the deregulation of the *ZEB1* and *ZEB2* (zinc finger E-box linking the genes homeobox 1 and 2) genes, involved in EMT, which subsequently leads to the development of metastases.

In our other work, a comprehensive assessment of the interaction between miRNAs, their target genes, and possible regulatory long non-coding RNAs in ovarian carcinoma was carried out. Analysis of the study results revealed a potential interaction between the *AURKA* (Aurora Kinase A) gene and miR-124 [27].

EMT is considered to be the early and most significant stage of cancer progression. This process is characterized by the transition of adhesive cells into motile cells and is regulated by miRNAs by reducing the expression level of genes responsible for adhesive or epithelial processes, such as E-cadherin, and increasing the expression of genes responsible for mesenchymal processes, such as N-cadherin. Normally, this process is reversible and is extremely important in the formation of various organs during embryonic development, as well as necessary for wound healing [4].

However, as the results of various studies show, violations of the regulation of the expression of several genes associated with this process can lead to the development and progression of cancers of various localizations, as well as affect resistance to different therapeutic markers. In particular, in a study conducted on breast cancer, it was shown that the interaction of miR-124 with the most significant genes regulating EMT, namely *ZEB1/2*, may be crucial for understanding the formation of cancer. Thus, the study showed that a decrease in the expression level of miR-124 in the tumor increased the expression level of the *ZEB2* gene, followed by the launch of EMT and the development of metastases. With a high level of miR-124 expression in the tumor, the reverse process was observed. Thus, using the example of triple-negative breast cancer, it was revealed that this gene is a direct target of miR-124 and is associated with both EMT and metastasis [28].

It should be noted that in the study of clear cell renal cell carcinoma, the synergy of miR-124 with another miR-203 miRNA was found. These regulators jointly suppressed the expression level of the *ZEB2* gene, which leads to the suppression of the proliferation and migration of tumor cells [29].

Importantly, in breast cancer, in addition to the EMT genes, another miR-124 target gene was identified. It is a signaling regulator and transcription activator STAT3, which is considered a specific oncogene for breast cancer. The authors note that miR-124 directly suppresses the activity of this gene, as a result reducing the proliferation, invasion, and migration of tumor cells [30].

Along with genomic and epigenomic rearrangements, significant changes occur in the metabolic system of the body with a tumor. A study of pancreatic cancer by a group of Chinese scientists has revealed that miR-124 inhibits the mechanism of metabolic transformation in tumor cells by suppressing the expression of the *MCT1* gene (monocarboxylate transporter type 1) and slows down cell growth [31].

Another target was identified in a study of liver cancer formation. The authors found that the suppression of miR-124 expression in the tumor led to increased expression of the *AKT1S1* (AKT1 Substrate 1) gene encoding the enzyme PRAS40. The reverse process was detected on cell lines. Based on these results, the authors indicated the diagnostic potential of this marker [32].

Recently, there has been an active study of the effect on the development of malignant transformation of immune control points. One of these points is the *PD-L1* gene (Programmed death ligand 1). This gene is a target for miR-124 and is also part of the STAT3 signaling pathway. According to the results of a study on colon cancer, miR-124 plays a huge role in tumors of this localization. It was shown that miR-124 was suppressed in the tumor, while the expression level of PD-L1 was high. The results on cell lines revealed the opposite effect. There was also a concomitant effect of lowering the expression level of several other genes, as *IL10* (interleukin 10), *IL2* (interleukin 2), *TNF-α* (tumor necrosis factor α), *TGF-β* (transforming growth factor beta), *IFN-γ* (interferon gamma). Additionally, an increase in the expression level of miR-124 decreased proliferation and termination of the cell cycle and triggered both pathways of apoptosis. The authors consider miR-124 as a therapeutic marker [33].

It should be noted that a target gene unrelated to immunity has been identified for this type of cancer. miR-124 enhances the expression of the *KiSS1* (Kisspeptin 1) gene reducing the proliferation of tumor cells, their invasion, and migration [34].

In the study of gastric cancer, a potential oncogene was identified, which is a direct target for miR-124. A study on cell lines showed that the *SRGAP1* gene (Slit-Robo GTPase-activating protein 1) enhances the proliferation, invasion, and migration of tumor cells. If this gene was suppressed because of a high level of miR-124 expression, the opposite effect was observed [35].

Recently, the studying of the influence of various factors on the properties of malignant transformation of stem tumor cells has become quite popular. This cell type is a precursor to the development of various types of tumors, changing its environment and adjusting the substrate for colonization, providing all further processes inherent. In their remarkable study, a group of Chinese scientists managed to identify interesting patterns. Based on the results of the work, miR-124 directly interacts with the *JAMA* (junctional adhesion molecule A) gene, which triggers the proliferation of tumor cells, promotes adhesion, and allows for plastic transition. The mutual regulation of this pair is of interest from the perspective of therapy, since a high level of miR-124 expression suppresses the expression of the target gene and thus inhibits the properties of tumor stem cells [36].

There should be also noted several studies in which the authors could trace entire regulatory pathways that trigger the processes of malignant transformation in tumors of various locations. Thus, in the study of squamous cell carcinoma of the esophagus, a pathway regulating the processes of growth and invasion in this type of cancer was identified. In addition to miR-124, the *DNMT1* (DNA methyltransferase 1) gene and the *BCAT1* (branched-chain amino acid transaminase) long non-coding RNA gene are involved in this pathway [37].

In another study on triple-negative breast cancer, the identified pathway was longer. Scientists have identified several genes and their regulators that are part of the large Wnt signaling pathway. The authors have approved miR-124 as a trigger factor for aberrant processes by targeting Axis inhibition protein 1 (Axin1). There was also a concomitant effect of changing the expression level of several other genes such as *BAX* (B-cell lymphoma-2 associated X), *BCL-2* (B-cell lymphoma-2), and *β-catenin* that are involved in the Wnt signaling pathway [38]. The same miRNA was also involved in STAT3/VEGF pathway, also detected in breast cancer. The authors claim that the direct target of miR-124 is cyanidin-3-glucoside (C3G) involved in the angiogenesis of breast cancer. This pair has been deemed a factor in the progression of breast cancer [39].

**Figure 1:**
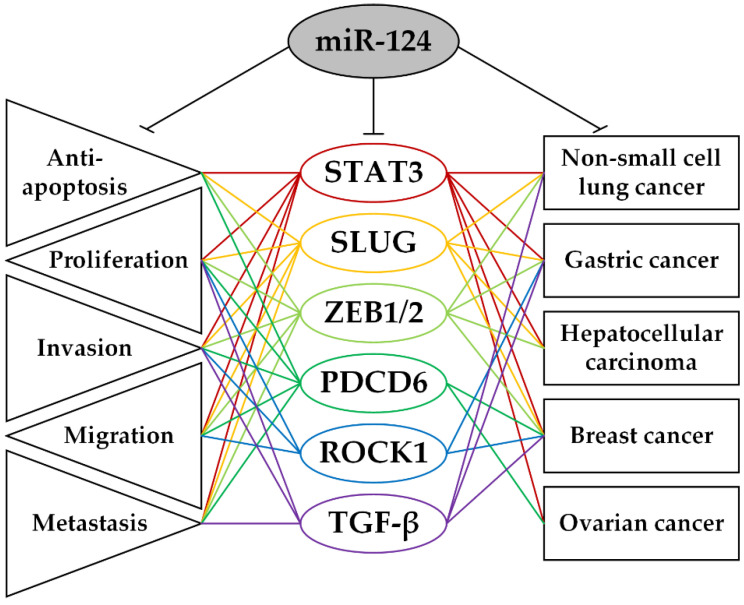
The scheme of differential regulation by miR-124: targets involved in the main biological processes in several common epithelial cancers. Central column—targets, left column—cell processes, right column—cancer types. The data from articles [3,4,16,28,30,40,41,42,43,44,45,46] were used.

By analyzing the above information, we chose a group of miR-124 regulated target genes (*PDCD6*, *ROCK1*, *SLUG*, *STAT3*, *TGF-β*, *ZEB1*), involved in the main biological processes in several common epithelial cancers, including lung, gastric, hepatocellular, breast and ovarian cancer, which is shown in Figure 1.

Separately, a recent bioinformatic screening of ovarian cancer databases made it possible to compile a complete network of interacting genes involved in the development and progression of ovarian cancer. According to the results of this study, the most significant targets for miR-124 in ovarian cancer may be genes: *CCNB1* (cyclin B1), *CEP55* (centrosome protein 55), *RACGAP1* (Rac GTPase-activating protein 1), *TPX2* (target protein for Xklp2), *UBE2C* (ubiquitin-conjugating enzyme E2C), *ZWINT* (ZW10-interacting kinetochore protein), and *CENPM* (centromere protein M). It is assumed that these genes can also be targets for miR-107, miR-34a-5p, miR-129-2-3p, and some others. The authors suggested that the identified interacting miRNA-mRNA pairs can serve as factors in the progression of ovarian cancer [47].

Bioinformatic database screening for bladder cancer followed by gene co-expression network analysis revealed six new genes (*PPARD*, *CST4*, *CSNK1E*, *PTPN14*, *ETV6*, and *ADRM1*) and several new miRNAs, including miR-124, as drivers in the development and progression of bladder cancer [48]. The predicted regulatory gene cascades involving miR-124 suggest even more multiple functions of this miRNA in epithelial cancers.

The results of all these studies confirmed miR-124 as a perspective biomarker for different cancer types.

It is known that miR-124 itself can be inhibited by hypermethylation of *MIR-124-1/-2/-3* genes, encoding miR-124 [5,6,7,26]. The important role of long and circular non-coding RNAs discovered in the last decade in the dysregulation of miR-124 and its targets has been established, which is the subject of the following sections of this review.

## 3. Long Non-Coding RNAs in Dysregulation of miR-124 Target Genes in Epithelial Cancers

In the last decade, the mechanism of regulation of protein genes involving not only miRNAs, but also other regulatory ncRNAs along the ncRNA/miRNA/mRNA scheme has received numerous confirmations. The interaction of miRNAs with target mRNAs and with regulatory ncRNAs requires the presence of the miRNA response elements (MRE) in sequences of both messenger RNAs and noncoding RNAs, such as long ncRNAs or circular ones [12,49,50]. It was shown that direct bindings are formed in ncRNA-miRNA and mRNA-miRNA pairs, and competition between ncRNA and mRNA for binding to miRNA is observed. This mechanism is called the competing endogenous RNAs (ceRNA) model [12,49,50]. Vast experimental data have been accumulated, supporting the involvement of lncRNA/miRNA/mRNA interactomes in the development and progression of cancer [51,52]. According to the ceRNA mechanism with the participation of suppressor miRNAs, such as miR-124, activation of target protein genes with oncogenic potential is usually observed. The lncRNAs involved in the inhibition of miR-124 also demonstrated oncogenic properties.

The discovery of new lncRNA/miRNA/mRNA interactomes is performed using a set of methods. Briefly, this is, firstly, a bioinformatics analysis that selects lncRNA/miRNA and miRNA/mRNA pairs from transcriptome databases, such as The Cancer Genome Atlas (TCGA), with a negative correlation of expression levels, but it is also necessary to identify a positive correlation between the levels of lncRNA and mRNA. Then, using the scanMiR tool (https://github.com/ETHZ-INS/scanMiR, accessed on 2 October 2022), the presence of a binding site for a given miRNA in the 3′-UTR mRNA and lncRNA sequences is checked [53]. Other approaches exist (e.g., [54]). Then, the bioinformatically identified transcriptome correlations are confirmed experimentally by analyzing the levels of RNAs of all three types in a representative set of cancer samples and evaluation of the expected expression level relationship (e.g., [55,56]). Further, functional studies are applied on cell lines of this type of cancer, which, in short, include transfection of synthesized RNAs into cells, artificial suppression or overexpression of triplet components, loss and gain of function, analysis of physiological changes in cell culture (as the level of proliferation and apoptosis, migration and cell invasion). Direct bindings between the components of the triplet are established using the luciferase test, RNA-binding protein immunoprecipitation (RIP) assay, and RNA pull-down assay. In some works, the authors confirmed the functional significance of these interactions not only in cell cultures but also with the use of model animals, usually immunodeficient mice (e.g., [55,56]). Some studies also elucidated the effect of lncRNA and interactome, or in other words, the lncRNA/miRNA/mRNA axis, on the survival rate and response to chemotherapy in patients.

In published research (PubMed on 11 September 2022), we have identified more than 40 such interactomes involving miR-124, which are summarized in Table 1. We have presented very briefly and compendiously data on the methods used for axis validation and on the functional role of triplets in the development or progression of the corresponding tumor types, as well as data on their clinical significance for patients (if available in the cited articles).

**Figure 2:**
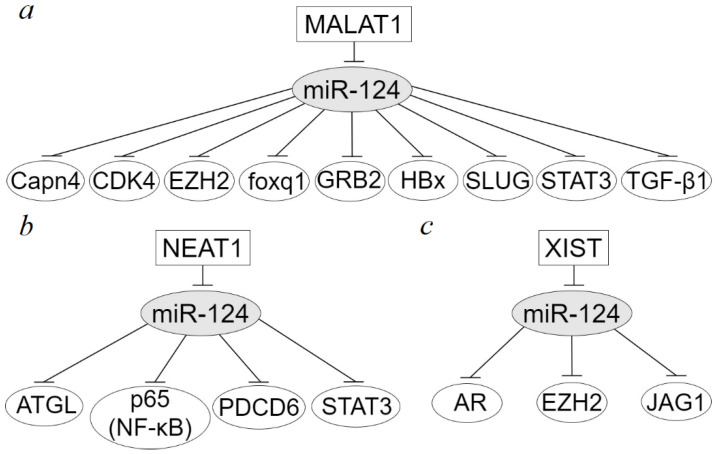
MiR-124 interactomes with the most abundant lncRNAs such as MALAT1 (**a**), NEAT1 (**b**), and XIST (**c**) in epithelial cancer.

As shown in Table 1, typical suppressor miR-124 is regulated, more precisely, inhibited in epithelial cancers exclusively by oncogenic lncRNAs, while miR-124 itself also targets mRNA of oncogenic proteins. The role of the most studied lncRNA MALAT1 (Metastasis-associated lung adenocarcinoma transcript 1) in the regulation of miR-124 is shown in nine studies conducted using primary tumors and cell lines or only cell lines of seven types of epithelial cancer: bladder transitional cell carcinoma, breast cancer, cervical cancer, gastric cancer, hepatocellular carcinoma, nasopharyngeal carcinoma, non-small cell lung cancer [40,73,74,75,76,77,78,79,80,97]. In these studies, mRNAs of the proteins Capn4, CDK4, EZH2, foxq1, GRB2, HBx, SLUG, STAT3, and TGF-β1 were identified as direct targets of miR-124, which is also shown in Figure 2a. Additionally, using a set of methods, the promoting effect of interactomes formed by MALAT1 on the progression of various types of cancer, including increased proliferation, migration, invasion, EMT, and, in contrast, suppression of apoptosis, was established. Experiments on mouse xenografts have shown that interactomes based on MALAT1, miR-124, and oncogenic proteins (as CDK4, EZH2, foxq1, HBx, and SLUG) also stimulate the growth and metastasis of tumors in vivo [74,75,76,78,79,97]. A decrease in the survival rate of patients under the influence of the MALAT1/mir-124 axes, which increase the level of expression of oncogenic proteins CDK4, foxq1, and SLUG [74,76,79,97], has been established. Depending on the target protein, the activation of several signaling pathways was revealed, such as E2F1 signaling (through activation of CDK4 protein expression), PI3K/Akt signaling, and stemness (through HBx activation), as well as TGF-β, SMAD, and ERK/MAPK signaling pathways (through activation of TGF-β1) [74,78,80].

Another highly represented in tumors and actively studied lncRNA NEAT1 (nuclear-enriched abundant transcript 1) was regulated in four types of cancer: breast cancer, hepatocellular carcinoma, nasopharyngeal carcinoma, and malignant thyroid nodules [81,82,83,84]. All these studies were conducted both on cell lines and on clinical samples from patients; xenografts of mice were used in two studies [81,82]. Four interactomes were identified: NEAT1/miR-124-3p/ATGL (adipose triglyceride lipase), NEAT1/miR-124-3p/p65 (NF-κB), NEAT1/miR-124/PDCD6 (programmed cell death 6), NEAT1/miR-124-3p/STAT3 (signal transducer and activator of transcription 3) [81,82,83,84], which is also shown in Figure 2b. These interactomes increased cell proliferation, migration, invasion, EMT and inhibit apoptosis in vitro and initiate tumor growth in immune-deficient mice in vivo. NEAT1/miR-124-3p/p65 (NF-κB) axis induces NF-κB signaling pathway in nasopharyngeal carcinoma [82]. NEAT1-mediated abnormal lipolysis promotes the growth of hepatocellular carcinoma cells [81]. Moreover, the activation of ATGL disrupts lipolysis in hepatocellular carcinoma cells, which is accompanied by the activation of DAG (diacylglycerol) + FFA (free fatty acids)/PPARα (peroxisome proliferator-activated receptor alpha) signaling [81].

LncRNA XIST (X-inactive specific transcript) inhibits miR-124 in three types of cancer: bladder cancer, laryngeal squamous cell carcinoma, and tongue squamous cell carcinoma, which activates AR (androgen receptor), EZH2 and JAG1 (jagged 1) proteins [94,95,96]. All three interactomes XIST/miR-124/AR, XIST/miR-124/EZH2, and XIST/miR-124/JAG1 are also shown in Figure 2c. These axes initiated by XIST are involved in promoting cell proliferation, migration, and invasion of three different cancer types as shown in vitro. Using tumor xenografts in nude mice, it was shown that the XIST/miR-124/EZH2 axis increased tumor growth in vivo in laryngeal squamous cell carcinoma [95]. Additionally, the XIST/miR-124/AR axis in bladder cancer has been shown to upregulate the expression of proliferation-associated factors, c-myc and p27, and metastasis-associated factors, MMP9 and MMP13 [94].

LncRNA HOXA11-AS (HOXA11 (Homeobox A11) Antisense RNA) also belongs to the most studied in the regulation of miR-124 and its protein-coding targets. Interactomes with HOXA11-AS have been identified in three types of cancer: hepatocellular carcinoma, gastric cancer, and non-small cell lung cancer, both on clinical samples and on cell lines [61,62,63]. The promoting effect of axes HOXA11-AS/miR-124/EZH2 (enhancer of zeste homolog 2 of polycomb group protein), HOXA11-AS/miR-124-3p/ITGB3 (integrin β3), and HOXA11-AS/miR-124/Sp1 (Sp1 transcriptional factor) on cancer cell proliferation, migration, invasion was shown in vitro. The most recent study [62] showed the effect of the HOXA11-AS/miR-124-3p/ITGB3 axis on growth and metastasis in vivo using mouse xenografts. Additionally, two studies have shown a decrease in patient survival under the action of axes that activate the expression of EZH2 and integrin β3 proteins [61,62].

Axes were also identified involving newer, recently discovered lncRNAs and targets, such as PTPRG-AS1 (protein tyrosine phosphatase, receptor type, G (PTPRG) Antisense RNA 1). This lncRNA is involved in two axes: PTPRG-AS1/miR-124-3p/CCND1 in lung adenocarcinoma and PTPRG-AS1/miR-124-3/LHX2 in nasopharyngeal carcinoma, which promote cell proliferation, cell cycle in vitro/in vivo, moreover, activation of LHX2 (LIM Homeobox 2) induces Notch pathway and reduces radiosensitivity [56,89].

The work that examined the role of suppressor lncRNA LINC01488 in the regulation of suppressor miR-124 and its target, mRNA of vimentin, in hepatocellular carcinoma [98] is somewhat aloof. Long intergenic non-coding RNA LINC01488 has been revealed as a key negative regulator of this cancer. The LINC01488/cyclin E/miR-124-3p/vimentin interactome does not directly bind the two suppressors, lncRNA LINC01488 and miR-124-3p. LINC01488 activates miR-124-3p through ubiquitination of cyclin E mediator, which can inhibit miR-124-3p. In cell cycle analysis, overexpression of LINC01488 inhibited G1 progression and suppressed S phase entry through effects on cyclin E function. Interestingly, the cyclin E mRNA level was not altered by LINC01488 regulation, indicating specific effects of LINC01488 on cyclin E at the translation level. Results from the RIP assay suggested that LINC01488 binds to cyclin E and decreases the expression of the protein via the ubiquitin-proteasome pathway. This is the only example of an interactome capable of following the function of miR-124-3p itself—to suppress proliferation, migration, invasion, and EMT of hepatocellular carcinoma cells in vitro, tumor growth, and metastasis in vivo. Additionally, this interactome decreases the overall and recurrence-free survival (OS, RFS) of patients. The LINC01488/cyclin E/miR-124-3p/vimentin interactome involves the ubiquitin-proteasome pathway.

Of interest are also works including recently published ones, in which the direct binding of lncRNA to miR-124, that can inhibit the expression of miR-124, and the effect of these interactions on cancer progression is clearly demonstrated, although target genes in these interactomes have not yet been identified. For example, increased expression of the oncogenic lncRNA NEAT1, which can bind and reduce the level of the suppressor miR-124-3p, has also been shown in ovarian cancer [99]. Elevated NEAT1 and decreased miR-124-3p levels are associated with advanced-stage and lymph node metastasis. NEAT1 can be stabilized by the RNA-binding protein HuR. Conversely, an increase in the miR-124-3p suppressor level can reduce the NEAT1 level and can be proposed for antitumor therapy [99].

In the tissues of the tongue squamous cell carcinoma (TSCC), an increase in the expression of the long intergenic non-coding RNA CASC15 (Cancer Susceptibility 15) and, conversely, a decrease in the level of miR-124 was revealed [100]. These changes are associated with poor overall patient survival. Additionally, it has been shown that overexpression of CASC15 can increase, whereas overexpression of miR-124 can reduce the ability of TSCC cells in cultures to migrate and invade [100].

A report on the oncogenic role of lncRNA SNHG16 (small nucleolar RNA host 16) in various cancer types and its involvement in many signaling pathways, such as TGF-β1/SMAD5, mTOR, NF-kB, Wnt, RAS/RAF/MEK/ERK, and PI3K/AKT, was published [92]. Moreover, this lncRNA serves as a sponge for several different miRNAs, among which the involvement of miR-124-3p was also revealed [92].

The effect of SNHG17, another member of the lncRNA family of small nucleolar RNA host genes (SNHGs), on breast cancer through direct binding of SNHG17 to miR-124-3p has been revealed. This study was conducted using rather reliable methods, such as luciferase reporter activity and RIP assays on cell cultures, as well as analysis of the expression in tissues of breast cancer patients and tumor growth in a xenograft model [101].

In non-small cell lung cancer, an inhibitory effect of MALAT1 on the miR-124 level and its association with EMT induction and cancer progression have been shown [102]. Inhibitory direct binding of MALAT1 and miR-124 has also been shown for cervical cancer in experiments on cell cultures and in vivo [103].

The interaction between lncRNA ZNF281 (zinc finger protein 281) and miR-124 predicted via IntaRNA 2.0 (http://rna.informatik.uni-freiburg.de/IntaRNA/Input.jsp, accessed on 2 October 2022) was confirmed using samples from gastric patients and cell cultures with the application of several standard approaches, such as transient cell transfections, qRT-PCR, loss- and gain-of-function, etc. The inhibition of miR-124, mediated by the oncogenic lncRNA ZNF281, promoted the migration and invasiveness of gastric cancer cells [104].

In a 2022 article, the interaction of lncRNA NEAT1 with miR-124-3p in ectopic endometrial cells was found to induce cell proliferation, migration, and invasion, which stimulates the malignant transformation of endometriosis and the development of endometrial cancer [105]. Another 2022 work showed direct inhibitory binding of long non-coding RNA DSCAM-AS1 (DSCAM antisense RNA 1) to miR-124 in hepatocellular carcinoma (HCC), inducing HCC cell proliferation [106].

Of interest, a purely bioinformatics analysis revealed another lncRNA, EMX2OS (EMX2 opposite strand antisense RNA), potentially involved in the regulation of miR-124, and two miR-124 target genes: *CALCA* (calcitonin-related polypeptide α) and *GABRG2* (γ-aminobutyric acid, subunit of γ2 type A receptor) [107]. Recurrent and non-recurrent laryngeal cancer sample datasets were downloaded from the Cancer Genome Atlas (TCGA) and the Gene Expression Omnibus database (GSE27020 and GSE25727). The new interactome EMX2OS/miR-124/CALCA, GABRG2 is presumably associated with molecular mechanisms of regulation of laryngeal cancer recurrent [107].

Thus, further studies must refine the targets in the presented data on incomplete lncRNA/mir-124 axes, and new detailed experimental work must validate the data of a purely bioinformatics analysis [107].

Summarizing the presented data, it can be argued that all complete interactomes that suppress the expression of miR-124 are involved in the mechanisms of activation of tumor cell proliferation, and most of these axes are involved in increased cell motility and invasion of epithelial cancer (Table 1). For 14 complete interactomes (HOTAIR/miR-124/ST8SIA4; HOXA11-AS/miR-124-3p/ITGB3; HOXA11-AS/miR-124/Sp1; KCNQ1OT1/miR-124-3p/TRIM14; LINC00240/miR-124-3p/DNMT3B; LINC00511/miR-124-3p/EZH2; LINC01410/miR-124-3p/SMAD5; MALAT1/miR-124/Capn4; MALAT1/miR-124/foxq1; SND1-IT1/miR-124/COL4A1; SNHG16/miR-124-3p/MCP-1; SP1- GCMA/miR-124/SLUG, SNAIL; UCA1/miR-124/JAG1; and XIST/miR-124/AR), involvement in EMT and/or development of metastasis in cancers of the corresponding localizations was revealed [43,59,62,63,64,65,67,70,73,76,90,91,93,94]. Moreover, the involvement of several interactomes (HOTTIP/miR-124-3p/HMGA2; NEAT1/miR-124/p65; MALAT1/miR-124/CDK4; MALAT1/miR-124/TGF-β1; MALAT1/miR-124/HBx; UCA1/miR-124/JAG1; PTPRG-AS1/miR-124-3/LHX2; OIP5-AS1/miR-124-5p/IDH2) into several signaling pathways, such as Wnt/b-catenin pathway, E2F1 signaling, TGF-β signaling, SMAD pathway, ERK/MAPK pathway, HIF-1α-pathway, Notch signaling, PI3K/Akt signaling, and cancer cell stemness has been established [60,74,78,80,82,87,89,93].

Additionally, several lncRNAs and their axes through the inhibition of miR-124 and activation of the expression of oncogenic proteins have shown clinical significance for cancer patients. Thus, 15 axes (HOXA11-AS/miR-124/EZH2; HOXA11-AS/miR-124-3p/ITGB3; LINC00511/miR-124-3p/EZH2; LINC00963/miR-124-3p/FZD4; lnc-1308/miR-124/ADAM15; MALAT1/miR-124/CDK4; MALAT1/miR-124/foxq1; MALAT1/miR-124-3p/SLUG; OGFRP1/miR-124-3p/LYPD3; OIP5-AS1/miR-124-5p/IDH2; PDIA3P1/miR-124-3p/TRAF6; SND1-IT1/miR-124/COL4A1; SNHG16/miR-124-3p/MCP-1; SP1-GCMA/miR-124/SLUG, SNAIL; UCA1/miR-124/JAG1) revealed poorer outcomes or shorter survival times for patients with various epithelial cancers [43,61,62,67,69,71,74,76,79,85,87,88,90,91,93].

Besides, the KCNQ1OT1/miR-124-3p/TRIM14 and PDIA3P1/miR-124-3p/TRAF6 axes increase chemoresistance, PTPRG-AS1/miR-124-3/LHX2 reduces radiosensitivity, and LINC00240/miR-124-3p/STAT3 inhibits natural killer T (NKT) cell cytotoxicity, which is critical for successful anticancer therapy [64,66,88,89].

## 4. The Circular Long Non-Coding RNAs in Dysregulation of miR-124 Target Genes in Epithelial Cancers

The decisive influence in the formation of the circRNA pool is made by a process called backsplicing. This is the excision by the spliceosome of the looping sections of the transcript. During splicing, introns are removed from the maturing transcript not as a linear fragment, but as a lasso. When cutting off the “tail” of the lasso, usually formed by inverted repeats of neighboring introns, circular RNA will be obtained. These are circular intronic long non-coding RNAs (ciRNAs). Additionally, in the maturing transcript, individual exons, one or more, can also loop out, and such loops can be excised by the spliceosome and covalently closed into a ring, forming exonic circRNAs (ecircRNAs). A region containing several introns and exons can also loop out (sometimes with subsequent continuation of splicing). This process produces exon-intron circRNAs (EIciRNAs). The exonic ecircRNAs accumulate predominantly in the cytoplasm, whereas the other two groups (intronic ciRNAs and exon-intron EIcircRNAs) accumulate predominantly in the nucleus. Relatively few circRNAs are believed to act as miRNA sponges, but there are those for which such interactions have been well demonstrated. First, they are, of course, exonic ecircRNAs. Because of the lack of a free 3′ or 5′ end, circRNAs have a long half-life and are more stable than linear RNAs. For this reason, circRNAs are considered highly effective biomarkers for various cancer types [108]. Table 2 summarizes the interactomes formed by circRNAs, miR-124, and messenger RNAs of target genes; the methods of their investigation and their functions in the carcinogenesis of epithelial tumors are given.

It can be seen from Table 2 that almost half, six out of 14, articles are devoted to the effect of circHIPK3 on miR-124 expression in various oncological diseases, although this phenomenon has been mostly studied in hepatocellular carcinoma. This ecircRNA is expressed at a high level in the cytoplasm of cells of various tissues (including lungs, heart, stomach, colon, brain). It is formed due to the circularization of the second exon of the *HIPK3* gene, flanked by introns containing complementary Alu repeats. circHIPK3 makes a significant contribution to the development of oncological diseases, the study of which can give a lot for their diagnosis and therapy. It can stimulate cell proliferation, autophagy, angiogenesis, EMT transition, inhibit pyroptosis, and cause the development of chemoresistance. Apparently, a significant part of these effects is associated with the influence on the expression of miR-124 and its targets, although the review [123] also mentions its interactions with other miRNAs.

When comparing Table 1 and Table 2, it can be noted that the effect of circRNAs on the development of epithelial cancers through the regulation of miR-124 is observed less frequently than the effect of lncRNAs, however, in most of the cited works, it has been convincingly proven by a wide range of methods. In the overwhelming majority of studies, this influence concerns the characteristics important for developing the disease and metastasis, such as the survival of cancer cells, their proliferative activity, and the ability to migrate and invade. It can be noted that in all studies, both circRNA and lncRNA directly interacting with miR-124 exhibit an oncogenic effect, which agrees well with the oncosuppressive effect of miR-124 itself.

In several studies, the influence of many circRNAs, such as circ MTHFD2, circHIPK3, and circPVT1, has been associated with a decrease in sensitivity to chemotherapy, which was studied both by determining resistance to specific drugs [116,117] and by measuring in vitro increase in the expression of the gene that causes multidrug resistance, as *MRP4* [112].

For ovarian cancer cells, the expression of cancer stem cell differentiation-related markers in vivo and in vitro was also studied, and it was concluded that circRNA, in particular circ_0026123, plays a significant role in maintaining cancer stemness [121]. In other words, the regulation of miR-124 by circRNA and lncRNA affects the same set of cancer cell characteristics, which indirectly indicates that miR-124 is correctly identified as the most significant miRNA in the interactomes described above.

The work [113] should be noted separately, where the effect of exosomes with miR-124 secreted by breast cancer cells was studied not on the cancer cells themselves, but on their environment (peripheral human endothelial cells). It was shown that miR-124 suppresses angiogenesis, whereas the effect of circHIPK3 on miR-124, in contrast, stimulates angiogenesis.

It should be noted that the role of lncRNA and circRNA in terms of modifying the effect of miR-124 on the microenvironment of cancer cells is not sufficiently disclosed in existing works. Thus, miR-124 significantly affects the immune response, including suppressing the development of various types of malignant tumors [124]. Additionally, exosomal miR-124 has been shown to suppress the transition of normal fibroblasts to cancer-associated fibroblasts in ovarian cancer [125]. The effect of lncRNA and circRNA on miR-124 activity in the tumor microenvironment may be the subject of further research.

We see that the set of those types of cancer, whose pathogenesis is significantly affected by the interaction of miR-124 with ncRNA, is generally very similar for lncRNA and circRNA. This indirectly confirms the thesis about the weakening of miR-124 expression as an important aspect of the pathogenesis of diseases, such as hepatocellular carcinoma, breast cancer, pancreatic cancer, gastric cancer, colorectal cancer, cervical cancer, and prostate cancer [3], and as an effective prognostic factor for these epithelial tumors [126]. Recall that miR-124 and the molecules interacting with it are considered promising targets for therapy in these diseases [3].

**Figure 3:**
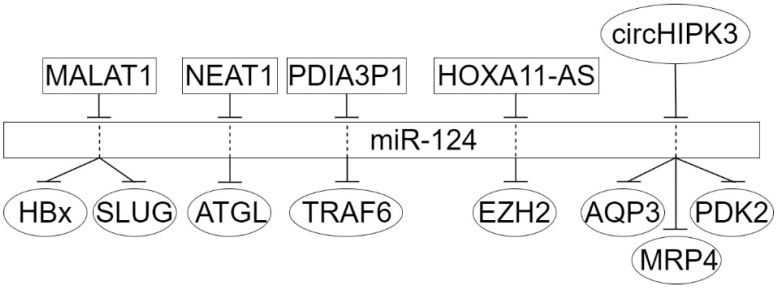
MiR-124 regulatory sub-network in hepatocellular carcinoma. LncRNAs are given inside the rectangles, circRNAs are in ovals at the top, protein targets are in ovals at the bottom.

**Figure 4:**
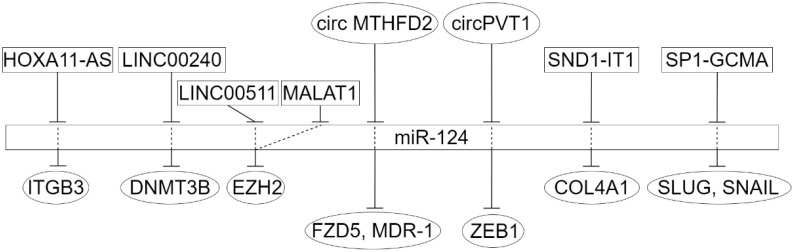
MiR-124 regulatory sub-network in gastric cancer. LncRNAs are given inside the rectangles, circRNAs are in ovals at the top, protein targets are in ovals at the bottom.

More problematic is the question of the targets of the influence of miR-124. Naturally, it should be borne in mind that not only for different types of cancer but also at its different stages, as well as for primary cancer/metastasis, these can be different targets. However, one can expect at least some agreement between the results of different authors. Thus, from the data of Table 1 and Table 2, it can be noted that for gastric cancer and hepatocellular carcinoma, on which the largest number of studies have been performed, ncRNA-miR-124 interactions and miR-124-mRNA interactions have been confirmed, and the significance of both of them for carcinogenesis has been proven (Figure 3 and Figure 4).

**Figure 5:**
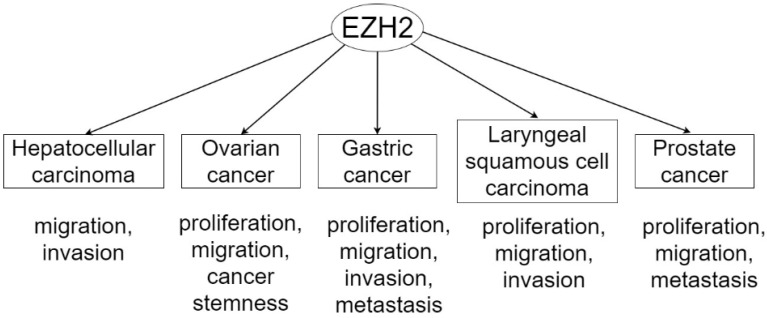
The role of EZH2 in the biological processes of carcinogenesis in five epithelial cancers.

However, in different studies, very different mRNA targets are indicated as significant, there is very little coincidence in them both within the same type of cancer and when comparing different types. The only exception is EZH2, which is characterized by numerous functions. An increase in the expression of EZH2 is associated with increased proliferation, migration, and invasion of cancer cells in various types of cancer: hepatocellular carcinoma, ovarian carcinoma, gastric cancer, laryngeal squamous cell carcinoma, and prostate cancer (Figure 5). Additionally, the *EZH2* gene has been identified in many different axes, both involving regulatory lncRNAs (HOXA11-AS, LINC00511, MALAT1, and XIST) and circRNAs (circ-TRPS1, hsa_circ_0026123), as can be seen by comparing Table 1 and Table 2. At present, ideas about the functions of EZH2 are being revised and supplemented. The canonical role of EZH2 is gene silencing by catalyzing trimethylation of lysine 27 histone H3 (H3K27me3) in a PRC2-dependent manner. However, evidence is accumulating that it can act as a transcriptional coactivator of genes involved in the development of various types of cancer, such as *c-Myc*, *cyclin D1*, *CXCR4*, *IL6*, *TNF*, *IGF1R*, *MMPs*, and *AR*.

Several studies have shown that biochemical modification of EZH2 induces dissociation from the PRC2 complex and converts EZH2 from a transcriptional repressor to a transcriptional activator. Its participation in interactions at a level other than transcription (binding of various RNAs, suppression of ubiquitination) is also assumed. For this reason, EZH2 is currently considered a novel and promising target for cancer therapy [127].

## 5. Conclusions

The typical suppressor miR-124 has a significant anti-oncogenic effect and it can inhibit the translation of at least fifty protein-coding targets. Among the targets regulated by miR-124, we could isolate at least six genes (*PDCD6*, *ROCK1*, *SLUG*, *STAT3*, *TGF-β*, *ZEB1*) common to several epithelial cancers, including lung, stomach, hepatocellular, breast, and ovarian cancers. Through the inhibition of oncoprotein translation, miR-124 induces apoptosis and cell cycle arrest, inhibits proliferation, invasion, EMT, metastasis, cancer cell stemming, chemoresistance, and improves the prognosis of patient survival, which has been shown in various types of cancer. Thus, miR-124 and its targets play a critical role in key biological processes in the development and progression of epithelial cancers.

Usually, the methylation of genes encoding miR-124 is considered first as a factor influencing the decrease in its expression. Without denying the significance of methylation of *MIR-124-1/-2/-3* genes, encoding miR-124, and the possibility of influencing its expression through the level of methylation, we consider it important to pay attention to alternative mechanisms.

Indeed, as has been clarified in the last decade, oncogenic lncRNAs and some circular RNAs form interactomes by the mechanism of competing endogenous RNAs (ceRNAs) according to the ncRNA/mir-124/mRNA scheme. These competitive interactions of mRNA and ncRNA (both lncRNA and circRNA) with miR-124 are possible if there are MRE sites for binding this miRNA in mRNA and regulatory ncRNA sequences. Moreover, oncogenic lncRNA and circRNA inhibit the suppressor miR-124, which, as a result, cannot effectively suppress the translation of its oncogenic protein targets.

More than 40 interactomes involving lncRNAs and miR-124 have been identified in epithelial cancers. LncRNAs MALAT1, NEAT1, HOXA11-AS, and XIST are the most represented in these axes. The largest number, nine interactomes, was formed with the participation of the widely studied lncRNA MALAT1 in epithelial cancer of seven types: bladder transitional cell carcinoma, breast cancer, cervical cancer, gastric cancer, hepatocellular carcinoma, nasopharyngeal carcinoma, and non-small cell lung cancer. mRNAs of the proteins Capn4, CDK4, EZH2, foxq1, GRB2, HBx, SLUG, STAT3, and TGF-β1 were identified as direct targets of miR-124 in these axes. LncRNA NEAT is involved in four interactomes, lncRNA HOXA11-AS is involved in three axes, and XIST is also involved in three axes. Interestingly, feedback loops are noted for many interactomes, such as for NEAT1/miR-124/STAT3. Axes involving new, relatively recently discovered lncRNAs, such as PTPRG-AS1/miR-124-3p/CCND1 and PTPRG-AS1/miR-124-3/LHX2, have also been identified. LncRNA PTPRG-AS1 through these axes promotes cell proliferation and cell cycle in vitro and in vivo, and activation of LHX2 induces the Notch pathway and reduces radiosensitivity.

Virtually all lncRNA interactomes inhibiting miR-124 and activating oncogenic proteins stimulate proliferation, inhibit apoptosis, and activate cell motility and cancer invasion, regardless of cancer type. A total of 14 axes, including lncRNAs and miR-124, are involved in EMT and/or metastasis in cancers. Moreover, eight axes are involved in key pathways: Wnt/b-catenin, E2F1, TGF-β, SMAD, ERK/MAPK, HIF-1α, Notch, PI3K/Akt signaling, and cancer cell stemness. Additionally, 15 axes showed a poor outcome or shorter survival time, and three axes decreased the chemo- or radiosensitivity of cancer patients.

To date, 14 interactomes involving circRNAs in the regulation of miR-124 and its targets have been identified. Nearly half of these include circHIPK3, which belongs to the exonic ecircRNAs found predominantly in the cytoplasm. This abundant circHIPK3 can stimulate cell proliferation, autophagy, angiogenesis, EMT, and chemoresistance, which has been shown in various cancers, although most of the work has been done on hepatocellular carcinoma. The influence of circ MTHFD2 and circPVT1 is also associated with a decrease in sensitivity to chemotherapy shown in gastric cancer.

It is important to emphasize that the luciferase reporter assay, RNA pull-down, and RIP assays were used as the most reliable methods for verifying the direct binding of miR-124 to ncRNAs (both, lncRNA and circRNA) and miR-124 to mRNAs in interactomes.

It should also be noted that exosomal miR-124 affects not only tumor cells, but also the microenvironment and significantly affects the immune response of cancer cells. However, there are few data on the analysis of the effect of lncRNAs and circRNAs on miR-124 activity in the tumor microenvironment, which requires further research.

As we have mentioned above, for gastric cancer and hepatocellular carcinoma, on which the largest number of studies have been performed, very different mRNA targets are indicated as significant in different studies. The only exception is EZH2, for which involvement in interactomes with both, lncRNA and circRNA, was shown in five epithelial cancers—hepatocellular carcinoma, ovarian carcinoma, gastric cancer, laryngeal squamous cell carcinoma, and prostate cancer. The *EZH2* gene has been identified in many miR-124 axes with four lncRNAs (HOXA11-AS, LINC00511, MALAT1, and XIST), and with two circRNAs (circ-TRPS1, circ_0026123). The multifaceted biological functions of this oncogenic protein and its involvement in all the most important processes of oncogenesis, such as proliferation, migration, invasion, metastasis, and increased stemming of cancer cells, have been noted. For this reason, EZH2 is currently considered a promising target for cancer therapy.

To summarize, the identification of protein target genes of miR-124 and regulatory interactomes involving long and circular ncRNAs reveals the multiple roles of miR-124 in the development and progression of epithelial cancers, which is realized through the ceRNA mechanism. Additionally, miR-124 and molecules interacting with it, demonstrate clinical significance as predictive markers, promising targets, and potential drugs for cancer therapy.

## Figures and Tables

**Table 1:** Interactomes lncRNA/miR-124/mRNA-Target in Epithelial Cancers.

LncRNA-Axis	Cancer	Methods of Analysis	Axis Functions	Ref.
HNF1A-AS1 /miR-124 /MYO6	colorectal cancer (40 patients, 4 cancer cell lines)	qRT-PCR, luciferase, RIP assays, Western blot, Transwell assay, glycolysis assessment	promotes cell proliferation, migration, invasion, activates glycolysis	[57,58]
HOTAIR /miR-124 /ST8SIA4	renal cell carcinoma (30 patients, 2 cancer cell lines)	qRT-PCR, luciferase assay, Western blot, FISH, Transwell assay, CCK-8, EdU, cell adhesion, colony formation, apoptosis, mouse xenografts	promotes proliferation, migration, invasion, decrease apoptosis in vitro; tumor growth and metastasis in vivo	[59]
HOTTIP /miR-124-3p /HMGA2	oral tongue squamous cell carcinoma (60 patients, 4 cancer cell lines)	qRT-PCR, luciferase assay, Western blot, MTT, Transwell assays, mouse xenografts	promotes proliferation, migration, invasion, tumor growth in vivo, Wnt/b-catenin signaling pathway	[60]
HOXA11-AS /miR-124 /EZH2	hepatocellular carcinoma (66 patients, 5 cancer cell lines)	qRT-PCR, cell transfection, loss-/ and gain-of-function, CHIP, RIP, Western blot, Transwell assay, Kaplan-Meier curve	mutual influence of triplet components, axis promotes migration, invasion, poor outcome	[61]
HOXA11-AS /miR-124-3p /ITGB3	gastric cancer (40 patients, 3 cancer cell lines)	qRT-PCR, luciferase reporter assay, western blot, Wound-healing assay, xenografts in nude mice, Kaplan-Meier plotter	promotes cell proliferation, migration, invasion in vitro, growth in vivo, metastasis, shorter OS, PFS	[62]
HOXA11-AS /miR-124 /Sp1	non-small cell lung cancer (78 patients, 4 cancer cell lines)	qRT-PCR, Western blot, luciferase reporter, RIP assays, cell proliferation, invasion assays	promotes invasion, proliferation, larger tumor size, lymph node metastasis	[63]
KCNQ1OT1 /miR-124-3p /TRIM14	tongue squamous cell carcinoma (60 patients, 2 cancer cell lines)	qRT-PCR, dual-luciferase, RIP, RNA pull-down assays, Western blot, MTT, Transwell assays, xenografts	promotes migration, invasion, EMT, in vitro/in vivo, cisplatin resistance	[64]
LINC00240 /miR-124-3p /DNMT3B	gastric cancer (48 patients, 2 cancer cell lines)	qRT-PCR, luciferase, AGO2-RIP assays, Western blot, migration, invasion assays, EMT-markers, xenografts in nude mice	promotes cell proliferation, invasion, migration, EMT in vitro, tumor growth in vivo	[65]
LINC00240 /miR-124-3p /STAT3	cervical cancer (167 patients, 5 cancer cell lines)	qRT-PCR, luciferase, RIP, RNA pull-down assays, Western blot, RNA FISH, CCK-8, Transwell, colony formation, tumor xenografts, cytotoxicity, T-cell conjugate assays	promotes cancer progression, cell proliferation, migration, invasion in vitro, in vivo; inhibits cytotoxicity of NKT cells via STAT3/MICA	[66]
LINC00511 /miR-124-3p /EZH2	gastric cancer (80 patients, 4 cancer cell lines)	qRT-PCR, dual-luciferase assay, Western blot, CCK-8 assay, Transwell assay, Kaplan-Meier	promotes proliferation, invasion, migration in vitro, tumor growth, metastasis in vivo, lower OS	[67]
LINC00511 /miR-124-3p /PDK4	gastric cancer (5 cancer cell lines)	qRT-PCR, luciferase, RIP, RNA pull-down assays, Western blot, caspase-3, CCK-8 assay, colony formation assay	promotes proliferation, inhibits apoptosis in vitro	[68]
LINC00963 /miR-124-3p /FZD4	colorectal cancer (84 patients, 4 cancer cell lines)	qRT-PCR, dual-luciferase assay, Western blot, CCK-8, Transwell, radioimmunoprecipitation assays, Kaplan-Meier survival curves	promotes cell proliferation, migration, associated with high TNM stage, shorter 5-year survival time	[69]
LINC01410 /miR-124-3p /SMAD5	cholangiocarcinoma (50 patients, 6 cancer cell lines)	qRT-PCR, luciferase, RNA pull-down, RIP, CCK8, colony formation, Transwell assays, Western blot	promotes cell proliferation, migration, invasion, colony formation ability, EMT	[70]
lnc-1308 /miR-124 /ADAM15	non-small-cell lung cancer (40 patients, 4 cancer cell lines)	human lncRNA microarray assay, qRT-PCR, miRIP, luciferase reporter assays, immunoblotting	promotes cell proliferation, invasion, poorer patient outcome	[71]
lnc-cCSC1 /miR-124-3p /CD44	colorectal cancer (4 cancer cell lines)	qRT-PCR, luciferase test, Western blot, CCK-8, colony formation, EdU staining, flow cytometry	promotes cell proliferation, inhibits apoptosis in vitro	[72]
MALAT1 /miR-124 /Capn4	nasopharyngeal carcinoma (4 cancer cell lines)	qRT-PCR, target prediction, luciferase assay, loss/gain of function, western blot, MTT assay, Transwell chamber assay, EMT-related proteins expression	promotes proliferation, migration, invasion, EMT in vitro	[73]
MALAT1 /miR-124 /CDK4	breast cancer (40 patients, 7 cancer cell lines)	qRT-PCR, dual-luciferase assay, Western blot, cell viability, cell cycle analyses, mouse xenografts, Kaplan-Meier curves	promotes proliferation, cell cycle progression, E2F1 signaling in vitro, tumor growth in vivo, poorer OS	[74]
MALAT1 /miR-124-3p /EZH2	gastric cancer (2 cancer cell lines)	qRT-PCR, predicted binding sites for miR-124-3p, gain-/loss-of-function, Western blot, MTT assay, Would healing scratch assay, mouse xenografts	promotes cells proliferation, migration in vitro, cancer growth in vivo; H_2_ downregulated MALAT1, suppressed growth of cancer	[75]
MALAT1 /miR-124 /foxq1	bladder transitional cell carcinoma (56 patients, 2 cancer cell lines)	qRT-PCR, luciferase assay, Western blot, cell proliferation assay, Transwell migration assay, invasion assay, tumor xenografts, Kaplan-Meier curves	promotes proliferation, EMT, migration, invasion in vitro, tumor growth, metastasis in vivo, shorter survival	[76]
MALAT1 /miR-124 /GRB2	HR-HPV (+) cervical cancer (22 patients, 3 cancer cell lines)	qRT-PCR, predicted binding sites, dual luciferase assay, western blot, Transwell analysis, flow cytometry	promotes proliferation, migration, invasion, inhibits apoptosis in vitro	[77]
MALAT1 /miR-124 /HBx	hepatocellular carcinoma (20 patients, 1 cancer cell line)	qRT-PCR, dual-luciferase assay, Western blot, cell colony formation assay, mouse xenografts	promotes proliferation, migration, invasion in vitro, stemness, progression in vivo, PI3K/Akt signaling	[78]
MALAT1 /miR-124-3p /SLUG	hepatocellular carcinoma (30 patients, 2 cancer cell lines)	qRT-PCR, cDNA array, luciferase, RIP assays, Western blotting, scratch wound healing, Transwell chamber assays, mouse xenografts, Kaplan-Meier curves	promotes migration, invasion in vitro, tumor growth in vivo, poor differentiation, lower DFS	[79]
MALAT1 /miR-124 /STAT3	non-small cell lung cancer (5 cancer cell lines)	qRT-PCR, RIP, RNA pull-down, dual-luciferase, Western blot, CCK8, colony formation, apoptosis assays	promotes proliferation, colony formation, inhibits apoptosis in vitro	[40]
MALAT1 /miR-124 /TGF-β1	nasopharyngeal carcinoma (6-10B cell lines)	qRT-PCR, cell transfection, luciferase reporter assay, loss and gain of function, Western blot, cell counting Kit-8, cell wound healing assay, cell Matrigel invasion assay	promotes proliferation, migration, invasion, TGF-β signaling, SMAD pathway, ERK/MAPK pathway	[80]
NEAT1 /miR-124-3p /ATGL	hepatocellular carcinoma (40 patients, 4 cancer cell lines)	qRT-PCR, Western blot, immunohistochemistry, luciferase reporter assay, orthotopic mouse xenografts	promotes proliferation in vitro/in vivo, disrupt lipolysis, DAG+FFA/PPARα signaling	[81]
NEAT1 /miR-124 /p65 (NF-κB)	nasopharyngeal carcinoma (20 patients, 5 cancer cell lines)	qRT-PCR, luciferase, RIP, RNA pull-down assays, Western blot, CCK-8, Colony formation assays, Flow cytometry, xenograft mice	promotes proliferation, inhibits apoptosis in vitro, tumor growth in vivo, NF-κB signaling pathway	[82]
NEAT1 /miR-124 /PDCD6	malignant and benign thyroid nodules (98 patients, 2 cancer cell lines)	qRT-PCR, luciferase assay, Western blot, immunohistochemistry, shRNA, ROC analysis	differs malignant from benign thyroid nodules; promotes EMT, cell migration, invasion in vitro	[83]
NEAT1 /miR-124 /STAT3	breast cancer (31 patients, 3 cancer cell lines)	qRT-PCR, luciferase assay, western blot, MTT, colony formation assays, flow cytometry, cell cycle analysis	promotes proliferation, migration, cell cycle progression, inhibit apoptosis	[84]
OGFRP1 /miR-124-3p /LYPD3	non-small cell lung cancer (120 patients, 5 cancer cell lines)	qRT-PCR, RIP, luciferase assays, colony formation, apoptosis assays, Western blot, migration and invasion assays, Kaplan-Meier curves	facilitates cell proliferation, migration, invasion, inhibits apoptosis in vitro; patient poor OS, DFS	[85]
OGFRP1 /miR-124-3p /SARM1, SAMD2	prostate cancer (57 patients, 4 cancer cell lines)	qRT-PCR, luciferase, RIP assays, FISH, clone formation assay, Wound healing, Matrigel invasion, apoptosis analysis	promotes tumor growth, metastasis, inhibits apoptosis, associated with TNM stages, perineural invasion	[86]
OIP5-AS1 /miR-124-5p /IDH2	cervical cancer (89 patients, 6 cancer cell lines)	qRT-PCR, luciferase assay, RIP assay, FISH, immunofluorescence, Western blot, cell proliferation assay, cell clone test, mouse xenografts	promotes cell proliferation, in vitro/in vivo, Warburg effect, HIF-1α-pathway, poor 5-years OS	[87]
hMTR4 /PDIA3P1 /miR-124-3p /TRAF6	hepatocellular carcinoma (174 patients, 2 cell lines)	qRT-PCR, luciferase, RIP, RNA pull-down assays, gain-/loss-of-function, in vitro, mouse xenografts, Kaplan-Meier curves	promotes cell proliferation, chemoresistance in vitro/in vivo, NF-κB pathway, reduces RFS; hMTR4 degrades lncRNA PDIA3P1	[88]
PTPRG-AS1 /miR-124-3p /CCND1	lung adenocarcinoma (cell cultures)	qRT-PCR, RNA pulldown, luciferase, RIP assays, flow cytometry, FISH, mouse xenografts	promotes cell proliferation, cell cycle in vitro/in vivo	[56]
PTPRG-AS1 /miR-124-3 /LHX2	nasopharyngeal carcinoma (61 patients, 5 cancer cell lines)	qRT-PCR, RNA pull-down, luciferase assays, Western blot; microarray, CCK-8, flow cytometry assays, site-directed mutagenesis	promotes NPC cell proliferation, reduces apoptosis, radiosensitivity; activates Notch pathway	[89]
SND1-IT1 /miR-124 /COL4A1	gastric cancer (52 patients, 4 cancer cell lines)	qRT-PCR, luciferase assay, CCK-8, Transwell assays, immunoblotting, EMT-markers	promotes migration, invasion, TGF-β1-induced EMT, metastasis, poor outcomes	[90]
SNHG16 /miR-124-3p /MCP-1	colorectal cancer (120 patients, 4 cancer cell lines)	qRT-PCR, luciferase, RIP, RNA pull down assays, Western blot, MTT, Wound healing, Transwell invasion assays, EMT-markers, mouse xenografts, Kaplan-Meier curves	promotes cell proliferation, migration, invasion, EMT in vitro, tumor growth, metastasis in vivo, reduces survival	[91,92]
SP1-GCMA /miR-124 /SLUG, SNAIL	gastric cancer (72 patients, 2 cancer cell lines)	qRT-PCR, ChIP-assay, luciferase, RIP assays, western-blot, RNA FISH, EMT-markers, xenografts in nude mice, Kaplan-Meier curves	SP1 activates GCMA via promoter; facilitates migration, invasion, EMT, metastasis in vitro/in vivo, worse OS, DFS	[43]
UCA1 /miR-124 /JAG1	tongue cancer (67 patients, 2 cancer cell lines)	qRT-PCR, luciferase, RIP assays, immunoblotting, Transwell invasion assay, immunofluorescence staining, Kaplan-Meier curves	promotes TGFβ1-induced EMT, metastasis, Notch signaling, poorer OS	[93]
XIST /miR-124 /AR	bladder cancer (67 patients, 4 cancer cell lines)	bioinformatic analysis, qRT-PCR, luciferase assays, loss-of-function, MTT assay, Transwell assay, MMP9, MMP13 activity, Western blot	promotes proliferation, invasion, migration, tumor growth, metastasis; increases factors c-myc, MMP9, MMP13	[94]
XIST /miR-124 /EZH2	laryngeal squamous cell carcinoma (34 patients, 2 cancer cell lines)	qRT-PCR, luciferase assay, Western blot, lentiviral transfection, shRNA, cell and colonies counting, Transwell assay, tumor xenografts in nude mice	promotes proliferation, migration, invasion in vitro, tumor growth in vivo	[95]
XIST /miR-124 /JAG1	tongue squamous cell carcinoma (cancer cell cultures)	qRT-PCR, luciferase assay, Western blot, Chip-seq analysis, CCK-8, scratch test	facilitates cell migration, proliferation in vitro	[96]
ZFAS1 /miR-124 /STAT3	esophageal squamous cell carcinoma (136 patients, 5 cancer cell lines)	qRT-PCR, luciferase, RIP, RNA pull-down assays, Western blot, cell co-culture model, fluorescence-labeled exosomes, FISH, colony formation, Transwell assays, flow cytometry, scratch test, xenografts in nude mice	promotes proliferation, migration, invasion, inhibit apoptosis in vitro, ZFAS1-exo promotes tumor growth in nude mice	[55]

Notes: ADAM15—a disintegrin and metalloproteinase domain 15; ATGL—adipose triglyceride lipase; AR—Androgen receptor; Capn4—Calpain 4, calcium-activated neutral protease small subunit; CDK4—cyclin-dependent kinase 4; DNMT—DNA methyltransferase; EZH2—enhancer of zeste homolog 2 of polycomb group protein; foxq1—forkhead box q1; FZD4—frizzled 4, a transmembrane protein; GCMA—Gastric Cancer metastasis-associated lncRNA; GRB2—Growth factor receptor-bound protein 2 (also known as an adaptor protein involved in signal transduction/cell communication); HBx—hepatitis B virus X protein; HMGA2—high-mobility group AT-hook 2; HNF1A-AS1—hepatocyte nuclear factor 1 homeobox A antisense RNA 1; HOTAIR—Hox transcript antisense intergenic RNA; HOTTIP—HOXA transcript at the distal tip; HOXA11-AS—HOXA11 (Homeobox A11) Antisense RNA; ITGB3—integrin β3; JAG1—jagged 1; KCNQ1OT1—potassium channel subfamily Q member 1(KCNQ1) overlapping transcript 1; LHX2—LIM Homeobox 2, a member of the LIM family that consists of 2 finger domains of zinc; MALAT1—Metastasis-associated lung adenocarcinoma transcript 1; MCP-1—Monocyte chemoattractant protein 1; MICA—MHC class I-related chain A; MMP9/MMP13—matrix metalloproteinases 9/13; MYO6—Myosins of class VI; NEAT1—nuclear-enriched abundant transcript 1; PDCD6—programmed cell death 6; PDIA3P1—protein disulfide isomerase family A member 3 pseudogene 1; PPARα—peroxisome proliferator activated receptor alpha; PTPRG-AS1—protein tyrosine phosphatase, receptor type, G (PTPRG) Antisense RNA 1; SND1-IT1—Staphylococcal nuclease and Tudor domain-containing 1 intronic transcript 1; SNHG16—small nucleolar RNA host gene 16; STAT3—Signal transducer and activator of transcription 3; ST8SIA4—alpha-2, 8-sialyltransferase 4; TRAF6—tumor necrosis factor receptor-associated factor 6; TRIM14—tripartite motif containing 14; UCA1—urothelial cancer associated 1; XIST—X-inactive specific transcript; ZFAS1—ZNFX1 (Zinc Finger NFX1-Type Containing 1) Antisense RNA 1. ChIP—chromatin immunoprecipitation; CCK-8 assay—cell counting kit-8; EdU assay—5-Ethynyl-2′-deoxyuridine assay; FISH—Fluorescence in situ hybridization; miRIP—microRNA in vivo precipitation method; RIP—RNA-binding protein immunoprecipitation assay. DAG—diacylglycerol; EMT—epithelial-to-mesenchymal transition; FFA—free fatty acids; H_2_—molecular hydrogen; HR-HPV (+) cervical cancer—high-risk human papillomavirus-positive cervical cancer; NKT cell—natural killer T cell; shRNA—short hairpin RNA; DFS—disease-free survival; OS—overall survival; PFS—progression-free survival; RFS—recurrence-free survival.

**Table 2:** Interactomes circRNA/miR-124/mRNA-Target in Epithelial Cancers.

CircRNA-Axis	Cancer	Methods of Analysis	Axis Functions	Ref.
circDOCK1 /miR-124 /CCND1	thyroid cancer, 25 patients, 2 cell lines	qRT-PCR, Transwell assays, overexpression of circDOCK1, miR-124 mimic, Western blots	cell migration, invasion, JAK/STAT/AMPK pathway	[109]
circHIPK3 /miR-124 /AKT3	Esophageal squamous cell carcinoma, 32 patients, 4 cancer cell lines, 1 normal	qRT-PCR, knockdown of circHIPK3, miR-124 mimic, AKT silencing, colony formation assays, Transwell assay, bioinformatics tools and dual-luciferase reporter gene assay for all interactions, Western blot, circHIPK3 knockdown in xenografts	cancer cell proliferation, migration, EMT; tumor growth in xenografts	[110]
circHIPK3 /miR-124 /AQP3	hepatocellular carcinoma, 50 patients, 5 tumor cell lines, 2 hepatocyte cell lines	qRT-PCR, CCK-8, Transwell assays, silencing circHIPK3, overexpression and knockdown of miR-124, overexpression and knockdown of AQP3, Sanger sequencing of circHIPK3, bioinformatics tools and luciferase reporter assay for circRNA-miR and miR-mRNA interaction, Western blots and immunohistochemistry, knockdown of circHIPK3 in xenografts	cell proliferation, migration, xenograft tumor growth	[111]
circHIPK3 /miR-124, miR-4524-5p /MRP4	hepatocellular carcinoma, 19 patients, 4 tumor cell lines, primary hepatocyte cells	qRT-PCR, knockdown of circHIPK3, miR-124 and miR-4524-5p inhibitor and mimic, bioinformatics tools and dual-luciferase reporter gene assay for pairs circRNA-miRNA, circRIP, Western blots	multidrug resistance	[112]
circHIPK3 /miR-124 /MTDH	peripheral human endothelial cells (ECs) mediated by the breast cancer (BC) cells-derived exosomal circRNAs	cell viability and tube formation, bioinformatics tools and dual luciferase reporter assay, western blot, qPCR assays, knockdown and overexpression of CircHIPK3, rescue experiment in mice xenograft model	cell viability, angiogenesis, impact on the microenvironment	[113]
circHIPK3 /miR-124 /PDK2	hepatocellular carcinoma, 30 patients, 4 tumor cell lines, 1 human hepatic cell line	qRT-PCR, CCK-8, EdU kit, Transwell assay, knockdown and overexpression of circHIPK3, miR-124 mimics, PDK2 overexpression plasmid, bioinformatics tools and dual-luciferase reporter gene assay for all interactions, Western blots, knockdown of circHIPK3 in xenografts	cell proliferation, invasion, xenograft tumor formation	[114]
circHIPK3 /miR-124 /ROCK1, CDK6	gall bladder cancer, 3 tumor lines, primary cultures of tumor, normal cells	QRT-PCR, CCK-8 viability assay, BrdU ELISA assay, Clonogenicity assay, TUNEL apoptosis assay, knockdown and overexpression of circHIPK3, overexpression of miR-124, Western blots	cancer cell survival, proliferation, inhibition of apoptosis	[115]
circ MTHFD2 /miR-124 /FZD5, MDR-1	gastric cancer, MGC-803 and MGC-803/MTA resistant cell model	qRT-PCR, screening of differentially expressed circRNAs, CCK-8, bioinformatics tool, microarray analysis, knockdown or overexpression of circ MTHFD2, miR-124 mimics transfection, luciferase reporter assay for circRNA-miRNA, Western blotting	resistance to pemetrexed (MTA)	[116]
circPVT1 /miR-124 /ZEB1	gastric cancer, 30 PTX-sensitive and 30 PTX-resistant patients, 4 tumor cell lines, 1 normal gastric cell line	qRT-PCR, MTT assay, flow cytometry, Transwell assay, knockdown and overexpression of CircHIPK3 and ZEB1, mimic and anti-miR for miR-124, bioinformatics tools and dual-luciferase reporter gene assay for all interactions, Western blot, circPVT1 knockdown in xenografts	PTX resistance	[117]
circ-TRPS1 /miR-124 /EZH2	prostate cancer, specimens from 80 patients, 3 tumor cell lines, xenografts	high-throughput sequencing, RT-qPCR, FISH, immunohistochemistry, tumor sphere formation assays, cell proliferation assays, colony formation assays, Transwell assays, knockdown of circ-TRPS1, miR-124 inhibition, EZH2 overexpression, bioinformatics tools, luciferase reporter assays for all interactions, Western blots, circ-TRPS1 knockdown in xenografts	cell proliferation and migration, metastasis	[118]
circ-VIM /miR-124 /PD-L1	esophageal cancer, 20 patients, 4 EC cell lines, embryonic kidney 293T cell line, normal esophageal HET-1A cell line	RT-qPCR, CCK-8, wound healing, Transwell assays, LDH assay, CFSE staining, Annexin V/PI staining, histological analyses, knockdown of circ-VIM, mimic and anti-miR for miR-124, bioinformatic tools, luciferase reporter assays for all interactions, RNA immunoprecipitation, Western blots, xenografts	circ-VIM silence synergizes with sevoflurane reduces immune escape and multiple oncogenic activities	[119]
circRNA_100782 /miR-124 /IL6R, STAT3	pancreatic ductal adeno-carcinoma, 2 tumor cell lines, 1 embryonic kidney cell line	qRT-PCR, colony formation assay, knockdown of circRNA_100782, mimic and anti-miR for miR-124, knockdown of STAT3, luciferase assay for circRNA-miR interaction, circRNA_100782 knockdown in xenografts	cell proliferation and colony formation	[120]
circ_0026123 /miR124 /EZH2	ovarian cancer, 20 patients, 4 OC cell lines, 1 normal ovarian cell line	RT-qPCR, FISH, CCK-8 assay, Transwell assays, knockdown, bioinformatic tools, luciferase reporter assay, Western blots, xenografts	cell proliferation, migration, stemness	[121]
circ_0000502 /miR-124	hepatocellular carcinoma, 40 patients, 6 HCC + 1 normal cell lines	qRT-PCR, CCK-8, Transwell assays, circ_0000502 knockdown, miR-124 overexpression, luciferase reporter gene assay for circRNA-miRNA pair	proliferation, invasion, migration, decrease in apoptosis	[122]

Notes: AKT3—AKT Serine/Threonine Kinase 3; AQP3—Aquaporin 3; CCND1—cyclin D1; CDK6—Cyclin Dependent Kinase 6; EZH2—Enhancer Of Zeste 2 Polycomb Repressive Complex 2 Subunit; FZD5—WNT Seven-Transmembrane Receptor Frizzled-5; IL6R—Interleukin 6 Receptor; MDR-1—Multidrug Resistance Protein 1; MRP4—Multidrug resistance-associated protein 4; MTDH—Metadherin; PD-L1—Programmed Cell Death 1 Ligand 1; PDK2—Pyruvate Dehydrogenase Kinase 2; PTX—paclitaxel; ROCK1—Rho Associated Coiled-Coil Containing Protein Kinase 1; STAT3—Signal Transducer And Activator Of Transcription 3; ZEB1—Zinc Finger E-Box Binding Homeobox 1. circDOCK1—circular transcript of protein coding gene Dedicator Of Cytokinesis 1; circHIPK3—circular transcript of protein coding gene Homeodomain Interacting Protein Kinase 3; circ MTHFD2—circular transcript of protein coding gene Methylenetetrahydrofolate Dehydrogenase (NADP + Dependent) 2; circPVT1—plasmacytoma variant translocation 1, circular transcript; circ-TRPS1—circular transcript of protein coding gene of Tricho-Rhino-Phalangeal Syndrome Type 1 (Transcriptional Repressor GATA Binding 1); circ-VIM—circular RNA of vimentin gene.

## Data Availability

Not applicable.
